# Beneficial Effects of Citrus-Derived Polymethoxylated Flavones for Central Nervous System Disorders

**DOI:** 10.3390/nu13010145

**Published:** 2021-01-04

**Authors:** Kentaro Matsuzaki, Yasushi Ohizumi

**Affiliations:** 1 Department of Environmental Physiology, Faculty of Medicine, Shimane University, 89-1 Enya-cho, Izumo 693-8501, Japan; 2 Kansei Fukushi Research Institute, Tohoku Fukushi University, 6-149-1 Kunimigaoka, Aoba-ku, Sendai 989-3201, Japan

**Keywords:** polymethoxylated flavones, nobiletin, tangeretin, 3,3′,4′,5,6,7,8-heptamethoxyflavone, central nervous system disorders

## Abstract

The number of patients with central nervous system disorders is increasing. Despite diligent laboratory and clinical research over the past 30 years, most pharmacologic options for the prevention and long-term treatment of central nervous system disorders and neurodegenerative disorders have been unsuccessful. Therefore, the development of drugs and/or functional foods to prevent the onset of neurodegenerative disorders is highly expected. Several reports have shown that polymethoxylated flavones (PMFs) derived from citrus fruit, such as nobiletin, tangeretin, and 3,3′,4′,5,6,7,8-heptamethoxyflavone, are promising molecules for the prevention of neurodegenerative and neurological disorders. In various animal models, PMFs have been shown to have a neuroprotective effect and improve cognitive dysfunction with regard to neurological disorders by exerting favorable effects against their pathological features, including oxidative stress, neuroinflammation, neurodegeneration, and synaptic dysfunction as well as its related mechanisms. In this review, we describe the profitable and ameliorating effects of citrus-derived PMFs on cognitive impairment and neural dysfunction in various rat and murine models or in several models of central nervous system disorders and identify their mechanisms of action.

## 1. Introduction

The number of patients with neurodegenerative diseases and neurological disorders associated with dementia, such as Alzheimer’s disease (AD), Parkinson’s disease (PD), and cerebrovascular dementia, is increasing [1,2]. Currently, approximately 50 million people worldwide have dementia, and that number is expected to triple by 2050 [1,3]. Because AD is the most common form of neurodegenerative disease [1,4,5], the development of new treatments for AD is anticipated. The earliest pathological symptom of AD involves the accumulation of β-amyloid (Aβ) plaques in the brain [5,6]. Briefly, Aβ plaques reportedly start to form more than 20 years before the onset of AD symptoms [6]. About 10 years after the start of Aβ accumulation, tau hyperphosphorylation and aggregation lead to the formation of neurofibrillary tangles in the brain [6]. A few years later, mild cognitive impairment develops, and the onset of AD occurs several years after that [6]. Thus, the mechanism of AD onset is supported by the amyloid cascade hypothesis, which states that abnormal accumulation of Aβ is responsible for cognitive decline [6]. However, abnormal accumulation of Aβ/tau in the brain occurs long before AD onset, and thus far, no clinical trials have successfully treated patients after AD onset. Researchers have recently shown that neuroinflammation and oxidative stress in the brain play major roles in the development of AD [7,8]. Interestingly, Venegas et al. (2017) reported that the overactivation of microglia in the brain elicits an inflammatory response that triggers Aβ accumulation [9]. It has also been reported that age-induced oxidative stress promotes Aβ accumulation in the brain, which suggests that AD onset might be caused by neuroinflammation or oxidative stress that precedes plaque formation in the brain [10]. Furthermore, studies have reported that lifestyle-related diseases, such as diabetes, dyslipidemia, and obesity, increase the risk of developing AD [11,12,13]. Therefore, preventing neuroinflammation, oxidative stress, lifestyle-related diseases, and the accumulation of Aβ/tau in the brain is a promising strategy for the prevention and treatment of AD. Neurodegenerative diseases and neurologic disorders have some pathological similarities at the intracellular and molecular levels, including oxidative stress, inflammation, and cognitive decline [7,8,14]. It is widely accepted that oxidative stress and neuroinflammation contribute to the progression of not only AD but also other neurodegenerative and/or neurological disorders, such as PD, cerebrovascular dementia, epilepsy, and depression [2,14]. However, therapeutic agents for these diseases have not yet been elucidated. Consequently, the development of functional foods to prevent neurodegenerative diseases and neurological disorders is highly expected.

**Figure 1:**
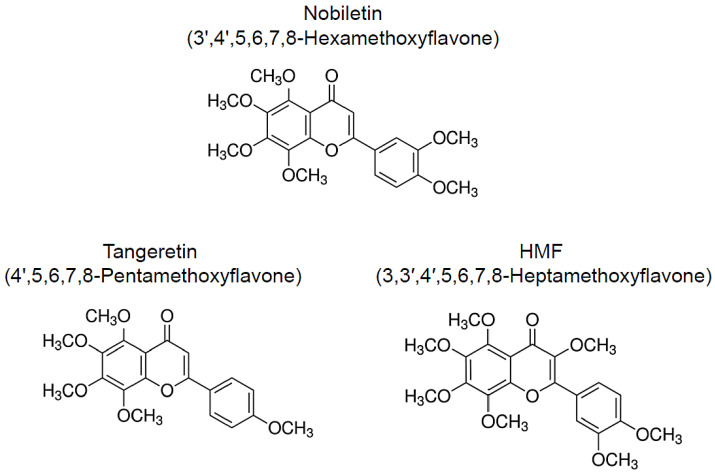
Chemical structure of citrus-derived polymethoxylated flavones, nobiletin, tangeretin, and 3,3′,4′,5,6,7,8-heptamethoxyflavone (HMF).

Numerous natural resources contain bioactive substances, which allows them to function as treatments and means of preventing geriatric and neurodegenerative diseases [14,15,16,17,18,19]. Citrus peels are a rich source of polymethoxylated flavones (PMFs) and have been widely used as a crude drug in traditional herbal medicines. The following compounds are major PMFs in citrus fruits (Figure 1): nobiletin (3′,4′,5,6,7,8-hexamethoxyflavone, C_21_H_22_O_8_), tangeretin (4′,5,6,7,8-pentamethoxyflavone, C_20_H_20_O_7_), and 3,3′,4′,5,6,7,8-heptamethoxyflavone (C_22_H_24_O_9_, HMF) [20]. Because of their anti-inflammatory and antioxidant effects, these citrus-derived PMFs have the potential to prevent neurodegenerative and neurological disorders [21,22,23,24,25,26]. In addition, these PMFs have shown beneficial effects against hyperlipidemia [27,28], obesity [29,30], diabetes [31,32], cardiovascular dysfunction [33,34], and cancer [35,36,37]. In this study, we focus on the neuroprotective and ameliorative effects of citrus-derived PMFs, nobiletin, tangeretin, and HMF, against central nervous system dysfunction in several rat and murine models of central nervous system disorders and related diseases as well as their mechanisms of action.

## 2. Nobiletin

Nobiletin is abundant in citrus fruits, such as *Citrus reticulata* and *Citrus depressa* [14,20]. Nobiletin is particularly abundant in the peel (outer pericarp) and albedo (middle pericarp), which are the pericarp parts [20]. The effectiveness of nobiletin in several model animals such as AD, PD, ischemic brain injury, oxidative stress, inflammation, and aging is herein mentioned. In addition, we have also investigated the effects of nobiletin on gene expression associated with oxidative stress and endoplasmic reticulum (ER) stress as well as protein activities associated with synaptic plasticity.

### 2.1. AD Model Animals

AD is one of the most commonly diagnosed neurodegenerative diseases [4,5]. The currently prevailing theory of AD pathogenesis is called the “amyloid hypothesis,” which suggests that AD is caused by pathological forms of Aβ accumulation in the brain (Aβ plaques) [4]. Two main Aβ peptides of different lengths (Aβ1-40 and Aβ1-42) are involved in AD [4,5]. The continuous infusion of Aβ1-40 into the lateral ventricle of the rat brain results in cognitive impairment [38]. Therefore, it is used widely as an animal model of AD [38]. The spatial cognitive function of this model was measured using the eight-way radial maze test [39]. The test results revealed that short- and long-term memory were impaired based on the increases in working memory error and reference memory error [39]. Nobiletin (10–50 mg/kg, intraperitoneally [i.p.]) was administered for 7 days before and after Aβ1-40 infusion into the right ventricle. Nobiletin was effective in preventing and improving Aβ1-40–induced memory impairment, as assessed by the eight-arm radial maze task [39]. Aβ is known to inhibit the phosphorylation of cAMP response element-binding protein (CREB) in neurons [40]. Although Aβ inhibited CREB phosphorylation in the primary hippocampal neurons, treatment with nobiletin significantly restored phosphorylated CREB levels [39]. Nobiletin may alleviate memory impairments by preventing the inhibition of CREB phosphorylation by Aβ.

The effect of nobiletin on Aβ1-42–induced spatial learning and memory impairment in mice has also been evaluated [41]. The administration of nobiletin (30 mg/kg orally [p.o.]) for 4 weeks prevented Aβ1-42–impaired spatial learning ability as assessed by the Morris water maze [41]. In addition, acetylcholinesterase activity in the cortex and hippocampus was improved by nobiletin. Furthermore, nobiletin significantly downregulated the Bax and cleaved caspase-3 protein expression and upregulated the B-cell lymphoma 2 (Bcl-2) and Bcl-2/Bax expression in the cortex and hippocampus of Aβ1-42–infused mice [41]. These results suggest that nobiletin induces a neuroprotective effect by regulating antiapoptotic mechanisms, including improved acetylcholinesterase activity in the cortex and hippocampus of the Aβ-infused animals.

Two main enzymes are involved in generating Aβ from amyloid precursor protein (APP): β-secretase (BACE1) and γ-secretase [42,43]. Another protein called tau may also contribute to neuronal death during AD when it becomes hyperphosphorylated and accumulates in neurofibrillary tangles [44]. APP-SL7-5 transgenic (Tg) mice overexpress a form of APP with two mutations: the London-type and Swedish-type [45]. These mice show Aβ accumulation in the hippocampus and cortex beginning at nine months of age and a marked accumulation of Aβ concomitant with cognitive impairment after 12 months of age [45]. Daily nobiletin (10 mg/kg, i.p.) administration from nine months of age for four months significantly improved fear-conditioned memory deficits [46]. Immunohistochemical analysis revealed that nobiletin reduces Aβ deposition in the hippocampus [46]. Furthermore, nobiletin significantly reduced guanidine-soluble Aβ1-40 and Aβ1-42 in the hippocampus [46]. Nobiletin may suppress the accumulation of Aβ in the brain and improve memory impairment in the hippocampus of the APP-SL7-5 Tg model mice.

The triple-transgenic mouse model of AD (3xTg-AD) have mutations in the genes for presenilin 1 (PS1), APP, and tau [47]. These mice exhibit AD-like pathologies such as plaque formation, neurofibrillary tangles, and cognitive dysfunction [47]. Administration of nobiletin (10–30 mg/kg, i.p.) for three months markedly improved short-term memory impairment in these mice [48]. Immunohistochemical analysis and enzyme-linked immunosolvent assay (ELISA) revealed that nobiletin administration significantly reduced levels of soluble Aβ1-40 in the hippocampus [48]. Nobiletin was shown to prevent short-term memory impairment by decreasing Aβ production and accumulation in the hippocampus of 3XTg-AD.

Aging is the most strongly-associated additive factor in neurodegenerative disease pathogenesis [49]. The deteriorated antioxidant function and the enhanced inflammatory response associated with aging are closely related to the development of neurodegenerative diseases, such as AD [49]. It is known that senescence-accelerated mouse-prone 8 (SAMP8) exhibits AD-like pathologies including abnormal expression of anti-aging factors as well as increased oxidative stress, neuroinflammation, Aβ deposits, tau hyperphosphorylation, ER stress, and cognitive dysfunction [50,51,52]. Behavioral studies showed that administering nobiletin (10–50 mg/kg, i.p.) to SAMP8 mice (~5 months old) for one month markedly improved object cognitive memory impairment and context-dependent fear memory impairment [53]. Glutathione (GSH) levels decreased in the cortex and hippocampus of SAMP8 mice; however, nobiletin administration caused significant improvements [53]. Nobiletin administration also decreased oxidized glutathione (GSSG) and carbonylated proteins (PC), which are increased by oxidative stress [53]. Furthermore, nobiletin significantly increases the activity of antioxidant enzymes, such as superoxide dismutase (SOD) and glutathione peroxidase (GPx) in the brains of SAMP8 mice [53]. Also, the hippocampus of SAMP8 mice had increased tau protein phosphorylation levels of serine (Ser) 202, threonine (Thr) 231, and Ser396 [53]. However, nobiletin significantly suppressed these increases [53]. These results suggest that nobiletin suppresses oxidative stress and tau phosphorylation while improving memory impairment in SAMP8 mice.

In AD patients, learning and memory are impaired by the degeneration and loss of cholinergic nerves in the cerebral cortex and hippocampus [54]. The OBX mouse is widely used as a model of AD-like dementia because it exhibits learning and memory deficits resulting from degenerating central cholinergic nerves after olfactory bulbectomy [55]. In OBX mice, intraperitoneal (50 mg/kg) and oral (50–100 mg/kg) nobiletin administration significantly ameliorated memory impairments according to the Y-maze test [56]. Also, nobiletin significantly suppressed cholinergic neurodegeneration in the hippocampus [56]. Nobiletin may also improve cognitive impairment by suppressing the degeneration of cholinergic nerves in the hippocampus of this model mouse.

Dysfunctional *N*-methyl-D-aspartate (NMDA) receptor-mediated neurotransmission is linked with AD-induced behavioral changes and cognitive deficits in addition to depressive disorders and suicidal behavior [57]. Administration of the NMDA receptor blocker (MK-801) to mice causes learning and memory deficits [58,59]. Seven days of continuous nobiletin (10–50 mg/kg, i.p.) administration significantly improved MK-801-induced memory impairment, as assessed by fear conditioning and passive avoidance tests [60]. Furthermore, MK-801 inhibited extracellular signal-regulated kinase (ERK) phosphorylation in the hippocampus, whereas nobiletin significantly restored levels of phosphorylated ERK [60]. Nobiletin may improve MK-801-induced cognitive deuteration by ERK activation in the hippocampus. 

### 2.2. PD Model Animals

PD is characterized by motor dysfunction due to the degeneration of dopaminergic neurons in the substantia nigra pars compacta [61]. Approximately 2–3% of adults older than 65 years are expected to be affected by PD [62]. It has been reported that the degenerative mechanism of dopaminergic neurons involves oxidative stress, neuroinflammation, mitochondrial dysfunction, protein aggregation and misfolding, excitotoxicity, apoptosis, and deficiency of trophic factors [61,62]. Thus, therapeutic strategies, such as the administration of antioxidants or anti-inflammatory drugs, activation of intracellular signal transduction pathways, and induction of neurotrophic factor expression, hold promise for the treatment and prevention of PD [63]. The intravenous administration of 1-methyl-4-phenyl-1,2,3,6-tetrahydropyridine (MPTP) selectively kills dopaminergic neurons, causing PD-like symptoms such as motor and cognitive dysfunction [64]. When taken up by dopaminergic neurons, 1-methyl-4-phenylpyridinium (MPP+), the active metabolite of MPTP, blocks mitochondrial complex I and thus deteriorates respiration, leading to the formation of superoxides [65]. In the MPTP model of PD, the administration of nobiletin (50 mg/kg, i.p.) for two consecutive weeks was found to improve motor impairment during the rotarod test and beam test [66]. Furthermore, nobiletin was found to significantly improve MPTP-induced cognitive impairment during the passive avoidance test and novel object recognition test [66]. In this model animal, degeneration of dopaminergic neurons in the striatum and CA1 region of the hippocampus as well as decreased levels of dopamine, cAMP-regulated phosphoprotein 32, and Ca^2+^/calmodulin–dependent protein kinase II (CaMKII) phosphorylation were confirmed. However, nobiletin significantly improved these functional declines [66]. Studies using several models of PD have also established that treatment with the neurotoxin MPP+ can damage dopaminergic neurons. Treatment with nobiletin (10 mg/kg body weight) preserved dopaminergic neurons in the substantia nigra of rats exposed to MPP+ [67]. Nobiletin also markedly suppressed microglial activation. This suggests that nobiletin might protect against MPP+-caused neurotoxicity via suppression of inflammation in the brain. Together, these results suggest that nobiletin protects dopaminergic neurons from MPTP− and MPP+-induced toxicity and may help prevent PD.

### 2.3. Ischemic Injury Models

Cerebral infarction is a cerebrovascular disorder that contributes to the development of vascular dementia [68]. During cerebral infarction, excessive generation of free radicals in the brain causes cellular damage, regardless of transient or continuous ischemia [68]. Because oxidative stress plays an extremely important role in the pathology of acute cerebral infarction, controlling oxidative stress is crucial for treating acute cerebral infarction [68,69]. Therefore, antioxidants, such as edaravone, are used widely in cerebral infarction treatment sites [70]. Carotid artery occlusion induces cerebral ischemia, which causes a high degree of cognitive impairment in mice. Nobiletin’s effect on memory impairment induced by cerebral ischemia in the occluded common carotid artery mouse model was examined using a passive avoidance test and a Y-maze test [71]. Continuous nobiletin (50 mg/kg, i.p.) administration for 7 days before and after occlusion of the carotid artery significantly improved associative and short-term memory impairment [71]. In the hippocampal CA1 region of mice with cerebral ischemia, the phosphorylation of CaMKII and the expression of microtubule-associated protein 2 were significantly reduced [71]. However, these reductions were attenuated by nobiletin treatment [71]. Nobiletin also attenuated the reduction in long-term potentiation due to ischemic injury [71]. Furthermore, the neuroprotective effect of nobiletin on rats with middle cerebral artery occlusion (MCAO) was also evaluated [72]. Pretreatment with nobiletin (10 and 25 mg/kg. i.p.) improved brain edema and impaired activities of Akt, CREB, brain-derived neurotrophic factor (BDNF), Bcl-2, and claudin-5 in the ischemic cortex [72]. In addition, nobiletin significantly improved brain edema, neurological deficit score and infarct volume 24 h post procedure [73]. Nobiletin also increased the Nrf2, HO-1, SOD1 and GSH levels, while decreased the levels of NF-κB, MMP-9 and malondialdehyde (MDA) [73].

Although blood flow is restored to ischemic brain tissue (ischemia/reperfusion), superoxide and hydroxy radicals are generated, which promotes cerebral edema and neuronal apoptosis [74]. In a mouse model of ischemia/reperfusion injury, nobiletin (30 mg/kg, i.v.) administration during occlusion and reperfusion of the rat middle cerebral artery suppressed cerebral edema and apoptosis [75]. In addition, nobiletin administration improved the motor dysfunction caused by cerebral ischemia/reperfusion [75]. The MCAO rat model was also established and treated with nobiletin [76]. Nobiletin treatment (5, 10, and 20 mg/kg, i.p.) notably improved neurological deficits, brain water content, and brain index in an MCAO model, accompanied by a decrease in the infarct area in the brain tissue [76]. Apoptosis induced by cerebral ischemia/reperfusion was also improved by nobiletin administration via the upregulation of Bcl-2 and downregulation of Bax and caspase-3 [76]. Nobiletin treatment also reduced the levels of the proinflammatory mediators tumor necrosis factor-α (TNF-α) and interleukin (IL)-6 and increased those of anti-inflammatory cytokine IL-10. Furthermore, the expression of phospho-p38 and mitogen-activated protein kinase-activated protein kinase 2 was reduced by nobiletin treatment in MCAO rats [76]. One study also reported that nobiletin enhances the neuroprotective effect of propofol on ischemia/reperfusion injury through the suppression of the Akt/mammalian target of rapamycin and nuclear factor kappa B (NF-kB) signaling cascade [77]. Because nobiletin exerts antioxidant and anti-inflammatory effects, this natural compound might have a protective effect against nerve cell damage caused by oxidative stress and/or inflammation generated by cerebral ischemia and reperfusion.

### 2.4. Lipopolysaccharide (LPS)-Induced Inflammation

Neurodegeneration due to excessive inflammation is involved in the development of AD and PD [7,8]. Numerous clinical and animal studies report that nonsteroidal anti-inflammatory drugs (NSAIDs) are effective for the prevention and treatment of AD and PD [78,79]. LPS is an inflammatory substance derived from Gram-negative bacteria that activates the mitogen-activated protein kinase (MAPK)/NF-κB pathway [79]. Activation of MAPK/NF-κB signaling activates microglia and stimulates the production of inflammatory cytokines, which may lead to cognitive impairment [79]. Nobiletin (100 mg/kg/day, p.o.) administration for six weeks prevented the increased expression of inflammatory mediators, such as nitric oxide (NO), TNF-α, interleukin IL-1, and IL-6 in an animal model of LPS-induced neuroinflammation [80]. Furthermore, nobiletin attenuated the LPS-induced activation of microglia and subsequent memory impairment [80]. The anti-inflammatory effect of nobiletin was also investigated in the BV2 microglia culture system [81]. Nobiletin (1–50 µM) treatment suppressed TNF-α and IL-1β levels [81]. LPS-induced phosphorylation of ERK, JNK, and p38MAPKs were also inhibited by nobiletin [81]. Although further research is needed about preventing neuroinflammation, nobiletin could be expected to prevent the neuroinflammation that leads to the development of PD.

### 2.5. Animal Model for Multiple Sclerosis

Multiple sclerosis is a complex chronic inflammatory and degenerative disorder of the central nervous system [82]. Demyelination and multiple sclerosis comprise the most common demyelinating condition. Nobiletin (50 mg/kg, i.p., two times/week) was administered in a cuprizone-administered demyelination model for three consecutive weeks. Nobiletin reduced the expression levels of myelin basic protein, a marker for mature oligodendrocytes, and increased immunoreactivity for platelet-derived growth factor receptor alpha, a marker for oligodendrocyte precursor cells (OPCs) [83]. Moreover, nobiletin enhanced the expression of proteolipid protein, a marker for mature oligodendrocytes and OPCs, and also increased the immunoreactivity to oligodendrocyte transcription factor 2 (OLIG2), a marker for OPCs, and their precursor cells. Nobiletin promoted the production of OPC in a model animal for demyelination [83]. Therefore, the effects of nobiletin on patients with demyelinating diseases such as multiple sclerosis may need to be studied in the future.

### 2.6. Chronic Unpredictable Mild Stress (CUMS)-Induced Synaptic Dysfunction and Depression-Like Behavior

Depression is a common among patients with AD, especially during the early and middle stages [84,85]. Nobiletin administration (25, 50, and 100 mg/kg, p.o.) significantly reduced the immobility time in both the tail suspension and forced swimming tests without accompanying changes in locomotor activity in the open-field test in mice [86]. The anti-immobility effect of nobiletin (50 mg/kg, p.o.) was prevented by the pretreatment of mice with a serotonin 5-HT(1A) receptor antagonist (WAY-100635, 0.1 mg/kg, subcutaneously [s.c.]), a serotonin 5-HT(2) receptor antagonist (cyproheptadine, 3 mg/kg, i.p.), an α(1)-adrenoceptor antagonist (prazosin, 62.5 μg/kg, i.p.), a dopamine D(1) receptor antagonist (SCH23390, 0.05 mg/kg, s.c.), or a dopamine D(2) receptor antagonist (sulpiride, 50 mg/kg, i.p.), respectively [86]. In addition, 5 weeks of nobiletin administration significantly ameliorated the CUMS-induced increase in serum corticosterone levels [86]. Furthermore, CUMS-induced loss of hippocampal BDNF, tropomyosin receptor kinase B (TrkB), and synapsin I was improved by nobiletin [86]. Nobiletin may have antidepressant effects through the improvement of the BDNF–TrkB pathway.

### 2.7. Various Mechanism of Neuroprotective Effect of Nobiletin

Central nervous system disorders have numerous pathological similarities at the sub-cellular and molecular levels, including oxidative stress, neuroinflammation, and memory impairment [7,8,87,88,89]. All these processes increase in the aging brain [90]. Increased oxidative stress in the brain due to aging is closely related to the development of central nervous system disorders, including AD and PD [91,92]. The effects of nobiletin on oxidative stress and ER stress have been examined in cultured cells such as the HuH-7 human hepatocarcinoma cells, 3Y1 rat fibroblasts, and SK-N-SH human neuroblastoma cells [93,94]. In the study, nobiletin markedly increased the expressions of endoplasmic reticulum stress response genes such as *DDIT3*, *TRIB,* and *ASNS* in the SK-N-SH human neuroblastoma-derived cell line. Meanwhile, the expression of *CCNA2*, a cell cycle control gene, and *TXNIP*, which encodes a thioredoxin-binding protein, decreased [93]. Furthermore, nobiletin suppressed tunicamycin-induced apoptosis as well as TXNIP protein expression upregulation [94]. These results indicate that nobiletin improves damage induced by oxidative stress disorder, which may explain its ability to improve cognitive function in various animal models of dementia. Also, it is plausible that nobiletin exerts neuroprotective effects by suppressing apoptosis caused by endoplasmic reticulum stress.

Learning and memory demand the formation of new neural networks and synthesis of new mRNA and proteins in the brain [95]. Intracellular signaling pathways are also involved in learning and memory [95]. The PKA/ERK/CREB cascade is one of the most important signaling pathway that regulates a wide variety of cellular processes, including proliferation, differentiation, learning and memory, development, and synaptic plasticity [96,97]. Memory formation depends upon CREB phosphorylation by kinases, such as PKA and ERK, and subsequent CRE-dependent transcription in hippocampal neurons [96,97]. Nobiletin significantly promoted CREB phosphorylation and CRE-dependent transcription as well as PKA activity and ERK phosphorylation in hippocampal primary neurons and the PC12 cells [98,99,100]. In addition, phosphodiesterase activity was significantly inhibited by nobiletin [98]. These results indicate that nobiletin promotes CREB phosphorylation and CRE-dependent transcription through PKA and ERK signaling, which enhances memory formation. Also, 4′-demethylnobiletin, a metabolite of nobiletin, also enhances PKA/ERK/CREB signaling in cultured rat hippocampal neurons [101]. This is one potential mechanism of action through which nobiletin and its derivatives improve cognitive function in various animal models of dementia.

The AMPA glutamate receptor contains four subunits (GluR1-R4) and is closely involved in synaptic plasticity, learning, and memory [102,103]. Increased PKA activity in the hippocampus, and subsequent Ser-845 phosphorylation on the AMPA receptor subunit GluR1 promotes the translocation of AMPA receptors to the cell membrane and induces LTP [103]. Nobiletin was found to potentiate AMPA receptor-mediated synaptic transmission at Schaer collateral-CA1 pyramidal cell synapses in mice hippocampal slices [104]. This potentiation induced by nobiletin was not accompanied by changes in the paired-pulse ratio, indicating the possible involvement of the postsynaptic mechanism [104]. Taken together, nobiletin may promote synaptic transmission in the hippocampus, through postsynaptic AMPA receptors at least partially by stimulating PKA-mediated phosphorylation of the GluR1 Ser-845. 

The NMDA-type glutamate receptor is a heterotetramer composed of the essential subunit NR1 and a regulatory subunit (NR2A-D) that provides diversity in function [105]. The distribution of NR1/NR2A and NR1/NR2B in the hippocampal CA1 region plays an important role in memory formation [106]. It was revealed that 24 h of nobiletin treatment in PC12 cells increased the gene expression of NR1 and NR2A by 1.4-fold and 1.7-fold, respectively [107]. Also, the NR2A gene showed a 1.3-fold increase at 3 h after the treatment [107]. An increase in NR2B expression was observed 6 h after treatment with nobiletin, and a 2.5-fold increase compared to the solvent control was confirmed after 24 h [107]. In particular, NR2B expression was markedly increased by nobiletin stimulation [107]. It is speculated that this is a result of the direct control of PKA/ERK/CREB activity by nobiletin because NR2B has a CREB binding site in its promoter region [107]. Nobiletin may enhance the expression of genes involved in learning and memory by activating the PKA/ERK/CREB signaling pathway, thus accelerating memory formation.

In the brains of AD patients, there are degeneration and loss of cholinergic nerves [54]. Furthermore, dysfunction of the olfactory nervous system appears during the early stages of AD [54]. There are five subtypes of mACh receptors (M1 to M5); Gq/11-coupled M1 is especially abundant in the hippocampus, cerebral cortex, striatum, and other regions [108,109]. It has also been reported that ChAT activity is significantly reduced in the brains of AD patients [110]. In PC12 cells, mACh receptor subtype M1 gene expression was shown to increase approximately 2.5-fold at 3 h after nobiletin stimulation. Its expression continued to increase for 12 h [111]. ChAT gene expression increased 1.7-fold and 2.1-fold at 3 and 6 h after nobiletin stimulation, respectively [111]. As described above, it has been revealed that nobiletin administration attenuates the decrease in ChAT protein levels observed in OBX mice. Nobiletin’s effects on the expressions of mACh receptor and ChAT may contribute to its ability to prevent and ameliorate cognitive dysfunction associated with cholinergic neurodegeneration. 

The Aβ fragment 25–35 (Aβ25–35) is also involved in the pathogenesis of AD [112,113]. Researchers have investigated the neuroprotective effect of nobiletin against Aβ25-35-induced neuronal cell damage [114]. Nobiletin protected Aβ25-35–induced apoptosis by restoring abnormal changes in intracellular oxidative stress markers, cell cycle, nuclear morphology, and activity of apoptosis-related signaling [114]. With regard to the anti-inflammatory responses, nobiletin markedly inhibited Aβ25–35-induced TNF-α, IL-1β, NO, and prostaglandin E_2_ (PGE_2_) production [114]. Moreover, nobiletin significantly inhibited Aβ-induced cyclooxygenase-2 (COX-2) and inducible NO synthase (iNOS) expression, which was attributed to the blockade of NF-κB p65 and phosphorylation of its inhibitor, IκB-α [114]. Moreover, this natural compound suppressed the phosphorylated c-Jun *N*-terminal kinase (JNK) and p38 levels without affecting the ERK1/2 level [114]. Nobiletin may also have the protective effects against Aβ25-35-induced neuroinflammation and subsequent cell death.

Recently, it has been reported that neprilysin can degrade Aβ [115,116]. However, neprilysin levels in the brain are known to decrease with advancing age and AD [117]. Likewise, it is expected that AD symptoms may be relieved by increasing the activity and expression levels of neprilysin in the brain. Interestingly, Fujiwara et al., found that nobiletin enhances the gene and protein expression of neprilysin as well as its enzymatic activity in cultured SK-N-SH cells [118]. Furthermore, nobiletin increased Aβ degradation through increased neprilysin expression [118]. Nobiletin also decreased the intracellular and extracellular levels of Aβ in iPS cells derived from AD patients that overproduce Aβ [119]. Nobiletin may enhance neprilysin expression and activity, thereby promoting Aβ degradation. Since nobiletin inhibits BACE1 activity [120], nobiletin may also decrease Aβ levels by interfering with its production. 

**Figure 2:**
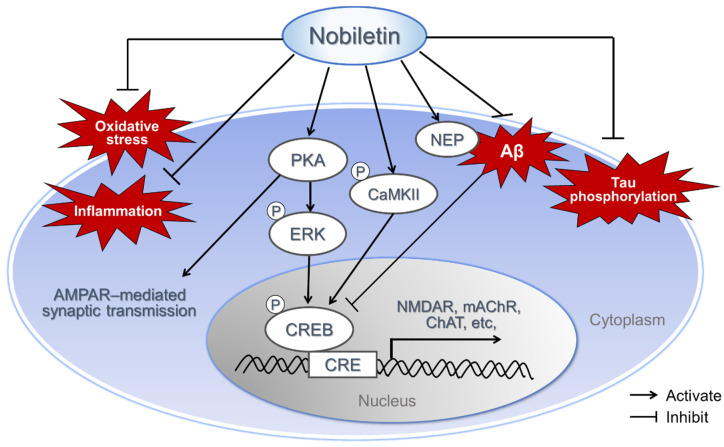
Potential neuroprotective effect of nobiletin. Nobiletin’s action mechanisms include various functions, such as antioxidant effects, anti-inflammatory effects, inhibiting Aβ production, increasing Aβ degradation, regulating gene expression through CRE-dependent transcriptional enhancement, inhibiting tau hyperphosphorylation, and regulating of synaptic plasticity. Aβ, β-amyloid; CaMKII, Ca^2+^/calmodulin–dependent protein kinase II; NEP, neprilysin; PKA, protein kinase A; ERK, extracellular signal-regulated kinase; CRE, cyclic adenosine monophosphate response element; CREB, CRE-binidng protein; NMDAR, *N*-methyl-D-aspartate receptor; mAChR, muscarinic acetylcholine receptor; ChAT, choline acetyltransferase; AMPAR, alpha-amino-3-hydroxy-5-methyl-4-isoxazolepropionic acid receptor.

Thus, nobiletin might be a potential candidate for amelioration of neurological diseases, such as AD, via antioxidative, anti-inflammatory, anti-Aβ pathology and regulation of signal cascades related to neurotransmission and CRE-mediated transcription (Figure 2).

## 3. Tangeretin

Tangeretin is a citrus PMFs with a structure similar to that of nobiletin. Tangeretin is found in the peel (albedo) of tangerine and other citrus fluits [20]. It appears to be a very noble phytochemical with many potential health benefits. Tangeretin is readily absorbed by tissues and has many beneficial properties such as neuroprotective actions and antidementia activity. The neuroprotective effect of tangeretin against PD, ischemic injury, epilepsy, chronic kidney disease (CKD), traumatic stress, oxidative stress and neuroinflammatory models were investigated.

### 3.1. PD Models

The effects of tangeretin in numerous PD animal models were investigated. Unilateral injection of 6-hydroxydopamine (6-OHDA), a synthetic catecholaminergic neurotoxin, into rats’ medial forebrain bundle significantly decreased the number of tyrosine hydroxylase-immunopositive (TH+) cells in the substantia nigra and declined the striatal dopamine content [121]. Tangeretin (20 mg/kg, p.o.) for 4 days improved the 6-OHDA–caused deterioration in TH+ cells and prevented the attenuation of dopamine content in the striatum [121]. It was also reported that tangeretin can pass through the blood–brain barrier and serve a neuroprotective effect in the brain.

Another study investigated that the neuroprotective effect of tangeretin in the prevention of neuroinflammation and improvement of dementia in MPTP-infused PD model rats [122]. Treatment with tangeretin significantly improved memory impairments and ameliorated motor functions. Histological analysis revealed the protective effects of tangeretin against MPTP-caused dopaminergic degeneration and hippocampal neuronal death. Tangeretin also attenuated the expression of the inflammatory mediators iNOS and COX-2 as well as those of the cytokines IL-1β, IL-2, and IL-6 [122].

ER stress induces a signaling reaction known as the unfolded protein response (UPR), which aims to restore proteostasis via the induction of adaptive programs when stress is chronic and/or unrepaired [123]. Abnormal levels of ER stress have been reported in the postmortem tissue of humans with PD as well as in most cellular and animal models of the disease [124]. Hashida et al. reported that tangeretin (10 mg/kg, p.o.) to MPTP-infused mice upregulated the expression of UPR-target genes in both dopaminergic neurons and astrocytes. Tangeretin treatment also facilitated neural cell survival [125].

In addition, Fatima et al. studied the effect of tangeretin on the symptoms of PD exhibited by PD model transgenic flies (*Drosophila melanogaster*) [126,127]. Tangeretin (5, 10, and 20 μM) was added to the flies’ diet, and the flies were allowed to feed on it for 24 days. At the same time, another set of PD flies were allowed to feed on a diet with 3–10 μM of L-DOPA. These authors studied the effect of tangeretin on dopamine content, lipid peroxidation, GSH, GST, PC, and monoamine oxidase activity, activity pattern, climbing ability, and the histopathology of the brain of PD model flies. Results indicated that exposure of PD flies to different doses of tangeretin was associated with a marked delay in the loss of climbing ability and an increase in dopamine content. Tangeretin also reduced the expression of various oxidative stress markers [126,127].

### 3.2. Cerebral Ischemia–Reperfusion Injury Model

Researchers have investigated the protective effect of tangeretin against ischemia–reperfusion injury in the rat [128]. Ischemia–reperfusion injury was induced in the brain via transient MCAO (2 h) and reperfusion (20 h). Tangeretin (5, 10, 20 mg/kg) significantly suppressed infarct volume, brain edema, brain water content, neurological score, and Evans blue leakage. Nobiletin also remarkably downregulated the inflammatory and proinflammatory cytokine levels of PGE_2_, iNOS, COX-2, IL-1β, toll-like receptor 4, interferon-γ, TNF-α, and IL-6 and the oxidative stress parameters SOD, GSH, GPx, catalase, glutathione reductase and MDA, in the serum and brain tissue of experimental rats. Thus, tangeretin could have neuroprotective and anti-inflammatory effects against ischemia–reperfusion injury in rats through suppression of inflammatory reaction and oxidative stress [128].

### 3.3. Epilepsy Model Rats

Epilepsy is a common neuronal disorder characterized by recurrent seizures [129,130]. The protective effect of tangeretin against neural apoptosis and seizure severity in pilocarpine (30 mg/kg, i.p.)-induced seizure model rats was investigated [131]. Oral administration of tangeretin (50, 100, or 200 mg/kg) improved the seizure scores and latency to first seizure of the rats and improved the pilocarpine-caused inhibition of PI3K/Akt pathway [131]. In addition, pretreatment of tangeretin reduced the number of TUNEL-positive cells in the in the hippocampal CA1 and CA3 regions. Tnangeretin also maintained the expressions of apoptosis-inducing factor in the mitochondria and the expressions of apoptosis-related proteins, e.g., Bcl-2, Bad, Bax and cleaved caspase-3. Moreover, seizure-induced elevations in the activities and expressions of matrix metalloproteinase (MMP)-2 and MMP-9 were modified by pretreatment of tangeretin [131]. Tangeretin may have a neuroprotective effect on pilocarpine-caused seizures through the activation of PI3K/Akt cascade and regulation of MMPs.

### 3.4. Nephrectomized Rats

CKD is a progressive loss of kidney function, and a significant health problem with limited therapeutic options [132]. Both oxidative stress and inflammatory responses have been associated in the pathology of CKD [132,133]. CKD patients frequently experience neurological complications that affect both the central and peripheral nervous systems, resulting in dementia [134]. The identification of effective treatment strategies has high clinical value in the therapy of CKD. In one study, five of six nephrectomized rats exhibited increased levels of MDA and reactive oxygen species [135]. Raised levels of cytokines IL-6 and IL-1β, and TNF-α, NO accompanied by activated NF-κB/TNF-α/iNOS signaling cascades were suppressed by tangeretin (50, 100 or 200 mg/kg, i.g.) [135]. In addition, cognitive dysfunctions and memory impairments observed in nephrectomized rats were rescued by tangeretin [135]. These data suggest that the antioxidant and anti-inflammatory effects of tangeretin ameliorated cognitive impairments in CKD model rats [135].

### 3.5. Posttraumatic Stress Disorder (PTSD) Model Rats

PTSD is a stress-related psychiatric/mental disease [136,137]. Research has shown that 14-day tangeretin (100 mg/kg, i.p.) treatment improves cognitive dysfunctions induced by a single prolonged stress episode mimicking PTSD induction in rats [138]. Tangeretin also improved the neurological abnormalities and the single prolonged stress-induced declines in dopamine and 5-HT levels in the hippocampus and amygdala [138]. These effects could be attributed in part to the induction of hippocampal genes encoding tyrosine hydroxylase and tryptophan hydroxylase 1 [139]. The ameliorating effects of tangeretin on memory and behavioral abnormalities associated with traumatic stress may need further study in the future.

### 3.6. Antioxidative and Antineuroinflammatory Effect of Tangeretin 

Antioxidative and neuroprotective activity of tangeretin against oxygen–glucose deprivation (OGD)-induced injury on human brain microvascular endothelial cells (HBMECs) was studied [140]. The researchers found that tangeretin prevented cell viability in response to OGD-induced injury and enhanced the viability of HBMECs. Tangeretin was able to enhance the activity of SOD and reduce the levels of reactive oxygen species and MDA as well as improve cell apoptosis in OGD-stimulated HBMECs. It was shown that these cytoprotective effects of tangeretin are related to the inhibition of the JNK signaling pathway [140].

On the other hand, the Nrf2 signaling cascade is one of the major pathways involved in the protection of cells against oxidative stress through upregulation of the expression of neuroprotective genes such as SOD [139]. Tangeretin was able to regulate the Nrf2 signaling pathway to exert antioxidant and anti-inflammatory activities [139].

To suppress microglial activation-mediated neuroinflammation has become a promising target for the development of medicates or functional foods to treat neurodegenerative diseases [141]. Shu et al. reported that tangeretin inhibits microglial activation implicated in the resulting neurotoxicity after LPS stimulation in cultured rat microglia and BV2 microglial cell models [142]. The results indicated that tangeretin suppressed the production of NO, PGE_2_;, TNF-α, IL-1β, and IL-6 in a dose-dependent manner. In addition, it inhibited the LPS-induced expression of iNOS and COX-2 in microglial cells [142]. Tangerine peel extract, which abundantly contains tangeretin, suppressed LPS-induced proinflammatory cytokine expression such as that of TNF-α, IL-1βand IL-6, and NO in the BV2 microglia culture system [143]. The anti-inflammatory effect of tangeretin was also investigated in rheumatoid synovial fibroblasts (RASFs) [144]. Tangeretin remarkably suppressed RASFs proliferation and downregulated the expression of MMP-1, MMP-3, and COX-2 levles and the phosphorylation of JNK, p38, and ERK. Tangeretin also reduced the elevated expression levels of PGE_2_ and NF-κB caused by IL-1 [144]. These results support further investigation of tangeretin’s therapeutic potential and molecular action mechanism regarding neuroinflammation and neurological diseases accompanying microglial activation.

## 4. HMF

HMF is another PMF found in citrus fruits [20]. Although it has relatively low content compared with nobiletin and tangeretin [20], evidences are accumulating on the neuroprotective effects of HMF. The effectiveness of HMF in numerous neural disorder models (e.g., cerebral ischemia, neuroinflammation, chronic stress, and epilepsy) has been examined. In addition, HMF has antioxidant, anti-inflammatory, and neurotrophic functions.

### 4.1. Cerebral Ischemia Mouse

The ERK/CREB cascade is one of the most important pathways in the regulation of synaptic plasticity as well as development of long-term memory [145,146]. After ischemia, HMF induced the phosphorylation of ERK1/2 and CREB in the hippocampus [147]. In addition, HMF significantly increased the expression of BDNF in the hippocampal dentate gyrus, and most BDNF-positive cells were also stained against glial fibrillary acidic protein [147]. HMF increased the number of doublecortin-positive neuronal precursor cells in the hippocampus [147]. These results suggest that HMF could promote BDNF production in astrocytes and enhance neurogenesis after brain ischemia, which may be caused by the activation of ERK1/2 and CREB.

Another study using a cerebral ischemia mouse model investigated the effects of HMF on protection against cognitive decline and neural cell death [148]. The authors found that the HMF administration for 3-day immediately after ischemic surgery improved against ischemia-induced cognitive impairment, rescued neuronal cell death, enhanced BDNF production, promoted CaMKII phosphorylation, and inhibited microglial activation in the hippocampus [148]. These results suggest that HMF has a neuroprotective effect for ischemic injury by causing BDNF upregulation via activation of the ERK/CREB cascade.

### 4.2. MK-801–Induced Memory Deficits and Locomotive Hyperactivity

HMF significantly induced the activation of ERK/CREB cascade in cultured cortical neurons [149]. Researchers have also investigated the administration of HMF in mice treated with the NMDA receptor antagonist MK-801 and found that it restored the MK-801–induced deterioration of spatial learning performance in the Morris water maze task [149]. HMFs may improve MK-801–induced cognitive dysfunction by activating ERK-related cascade in the cortex [149]. In addition, the improvement effect of HMF on MK-801-induced locomotive hyperactivity was served by phosphorylation of ERK1/2 in the hippocampus [150]. Interestingly, intraperitoneally injected HMF were immediately detected in mice brain. Moreover, the permeability to the brain tissues of HMF was significantly greater than that of other citrus PMFs such as nobiletin and tangeretin [150]. The permeation of these PMFs into mice brain correlated with their abilities to improve MK-801-induced behavioral abnormalities, indicating that HMF can cross blood-brain barrier and directly affectable to the brain [150]. This finding could provide clues to the structure–activity relationship of PMFs with antidementia and neuroprotective properties.

### 4.3. Antineuroinflammatory Effect

The effect of HMF on inflammation in the hippocampus was investigated using mice injected intrahippocampally with LPS [151]. HMF prevented LPS-induced body weight loss as well as microglial activation in the hippocampus [151]. In addition, HMF suppressed the mRNA levels of IL-1β and TNF-α and that of COX-2 in the hippocampus [151]. Ihara et al. also reported that HMF inhibited LPS-induced iNOS protein and mRNA expression by suppressing the activation of NF-κB and the phosphorylation of the p38 in rat primary astrocytes [152]. These results suggest that HMF has the ability to reduce neuroinflammation in the brain.

### 4.4. Stress-Induced Depression Models

One study investigated the antidepressive effects of HMF in mice injected subcutaneously with corticosterone at a dose of 20 mg/kg/day for 25 days [153]. HMF treatment ameliorated corticosterone-induced depression-like behavior, as evaluated by the forced swim and tail suspension tests [153]. In addition, corticosterone-caused BDNF depletions in the hippocampus were improved by HMF [153]. HMF administration also ameliorated the corticosterone-induced reductions in neurogenesis in the dentate gyrus subgranular zone accompanied by reductions in the expression levels of phosphorylated CaMKII and ERK1/2 [153].

The effect of HMF on the CUMS model was also investigated [154]. Researchers found that CUMS-induced depressive-like behavior was ameliorated by HMF [154]. HMF administration restored the CUMS-induced reduction in BDNF expression, decreased neurogenesis, and decreased levels of phosphorylated CaMKII in the hippocampus [154]. These effects were inhibited by the pretreatment of U0126, a MAPK inhibitor, suggesting that HMF exerts its effects as an antidepressant drug by inducing ERK activation and BDNF expression [154].

### 4.5. Neurotrophic Effect of HMF

Neurotrophic effect of HMF in vitro using rat C6 rat glioma cells was investigated [155]. HMF enhanced the cAMP level, ERK and CREB phosphorylation, and BDNF expression in C6 rat glioma cells [155]. In addition, HMF inhibited the phosphodiesterases PDE4B and PDE4D [155]. HMF induced BDNF upregulation was abolished by U0126. These findings suggest that HMF might exert its neurotrophic effects by inducing the expression of BDNF in C6 cells via the activation of cAMP/ERK/CREB signaling [155]. Given that HMF induce ERK activation in cultured cortical cells [149], this natural compound may affect both neuronal and glial cells in the brain.

Moreover, 5-hydroxy-3,6,7,8,3′,4′-hexamethoxyflavone (5-OH-HMF), an analog of HMF, was shown to efficaciously prompt neurite outgrowth of PC12 through the upregulation of growth-associated protein 43, a neural differentiation marker [156]. 5-OH-HMF also enhanced CREB phosphorylation, CRE-mediated transcription, and c-fos gene expression [156]. Moreover, both CRE transcription and neurite outgrowth induced by 5-OH-HMF were inhibited by the PKA inhibitor H89 [156]. Thus, 5-OH-HMF might also have a neuroprotective effect on neural disorders through regulation of gene expression via CRE-mediated transcription.

## 5. Shared Functions and New Research Perspectives

The three types of PMFs exerted neuroprotective and neurotrophic effects in various experimental models. These natural compounds share common mechanisms such as antioxidant and anti-inflammatory effects. The antioxidant and anti-inflammatory effects of PMFs may be an important mechanism of neuroprotective effect in various neurological models. On the other hand, PMFs seem to activate neural function through activation of several intracellular signal cascade and gene expression. Therefore, structure-activity relationship studies may be needed in order to understand the precise mechanism of action of the neuroprotective effects of PMFs.

We further mention herein the facts that there is one exciting discovery of PMFs’ function which deserve additional discussion. Circadian rhythms are biological activity rhythm driven by internal circadian clocks and are a fundamental mechanism to regulate various pathways and pathophysiology [157]. Circadian disruption induces the development of numerous diseases, including obesity, metabolic syndrome, neuroinflammation and cognitive impairment [157,158,159]. In addition, disruption of circadian rhythms is a common occurrence in elderly individuals, and is more severe in patients with neurodegenerative diseases, such as AD and PD [158,159]. Interestingly, nobiletin, and to a certain degree also tangeretin, has been reported to activate circadian rhythms, and confer protection against metabolic disease, aging and delirium [160,161,162,163,164,165]. The regulation of circadian rhythms by PMFs may partly be involved in the improvement of neuronal function in several neurological disease model animals.

## 6. Conclusions

**Figure 3:**
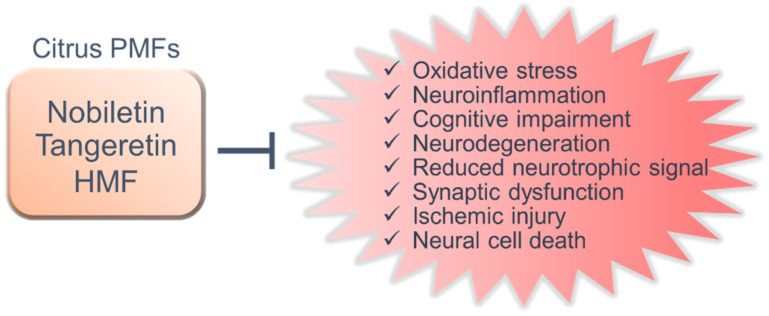
Potential therapeutic mechanism of nobiletin, tangeretin and 3,3′,4′,5,6,7,8-heptamethoxyflavone (HMF) for central neurological diseases.

In this paper, we reviewed the neuroprotective effect of citrus PMFs, nobiletin (Table S1), tangeretin (Table S2) and HMF (Table S3), and highlighted their potential action mechanisms. PMFs significantly prevent and/or improve cognitive dysfunction and motor dysfunction in animal models. These action mechanisms involve diverse functions, such as antioxidant effects, anti-inflammatory effects, inhibition of Aβ pathology, suppression of neurodegeneration and neuronal cell death, regulation of neurotrophic signals and synaptic plasticity (Figure 3). Furthermore, citrus PMFs exert antidementia effects after oral, subcutaneous and intraperitoneal administration in animal models of neurodegenerative diseases and neuronal disorders. Nobiletin, tangeretin, HMF, and its bioactive metabolites can also cross the blood–brain barrier [150,166,167]. PMFs are generally quite safe, which is a major advantage. It has been shown that chronic administration of the extract of *Citrus reticulata Blanco*, *Citrus reticulata or Citrus sinensis*, which contain high concentrations of PMFs, have no harmful effects on animals [168,169], and humans [170,171]. 

Citrus peels and/or extract have been reported to have various beneficial effects on humans, e.g., body weight control [171], promote cardiovascular health [172], improve hepatic steatosis [173] and cancer prevention [174,175]. Although consisting of only a few cases, one clinical study has demonstrated that PMF-rich citrus peel extract prevents the progression of cognitive dysfunction in AD patients on donepezil therapy [176]. Based on this evidence, it is important to develop functional foods or drugs that could prevent or ameliorate central nervous system disorders.

## Supplementary Materials

The following are available online at https://www.mdpi.com/2072-6643/13/1/145/s1, Table S1. Neuroprotective and beneficial effect of nobiletin in several experimental models for neurological disorders; Table S2. Beneficial effect of tangeretin in neurological disease models; Table S3. Multiple effects of 3,3′,4′,5,6,7,8-heptamethoxyflavone (HMF) in neurological disease models.
