# Supplementary material for: Beneficial Effects of Citrus-Derived Polymethoxylated Flavones for Central Nervous System Disorders
Source: Nutrients. 2021 Jan 4;13(1):145. doi: 10.3390/nu13010145 (PMC7824236; doi:10.3390/nu13010145)
Supplement: Supplementary file 1 [file nutrients-13-00145-s001.pdf]

Table S1. Neuroprotective and beneficial effect of nobiletin in several experimental models for neurological disorders.

Experimental model	Biological activity	Dosage	Reference
Aβ1-40-infused rats	Nobiletin improved Aβ1-40-impaired working memory and reference memory assessed by 8-arm radial maze.	10-50 mg/kg (i.p.) for 7 days before and after Aβ1-40 infusion	[39]
Aβ1-42-infused mice	Nobiletin ameliorated spatial learning and memory deficits evaluated by Morris-water maze and passive avoidance task. AchE activity, Bcl-2 and Bcl-2/Bax level in the cortex and hippocampus were also improved.	30 mg/kg (p.o.) for 4 weeks after Aβ1-42 infusion	[41]
APP-SL7-5 Tg mice	Nobiletin preserved fear-conditioned memory assessed by passive avoidance task and reduced both Aβ1-40 and 1-42 level in the hippocampus.	10 mg/kg, (i.p.) from 9 months of age for 4 months.	[46]
3xTg-AD mice	Nobiletin prevented short-term memory and recognition memory evaluated by Y-maze and Morris water maze. Hippocampal Aβ1–40 and ROS level was reduced.	10-30 mg/kg (i.p.) for 3 months	[48]
SAMP8 mice	Nobiletin improved object cognitive memory and context-dependent fear memory assessed by Nobel object recognition task. Oxidative stress markers and Tau hyper-phosphorylation were suppressed.	10-50 mg/kg (i.p.) for 1 month	[53]
Olfactory bulbectomized mice	Nobiletin prevented memory impairments evaluated by the Y-maze, and cholinergic neurodegeneration in the hippocampus.	50 mg/kg (i.p.) or 50-100 mg/kg (p.o.) for 11 days from 3-day after OBX surgery	[56]
MK-801-infused mice	Nobiletin improved memory impairment, as assessed by fear conditioning and passive avoidance task, and ERK phosphorylation in the hippocampus.	10-50 mg/kg (i.p.) for7 days	[60]
MPTP-infused mice	Nobiletin improves motor impairment during the rotarod test and beam test, and ameliorated cognitive impairment during the passive avoidance test and Novel object recognition test. DARPP-32 and p-CaMKII expression level in the hippocampus was also improved.	50 mg/kg (i.p.) for 2 consecutive weeks	[66]

MPP+ -treated rats	Nobiletin maintained dopaminergic neurons, inhibited microglial activation and preserved the expression of GDNF in the substantia nigra.	10 mg/kg (i.p.) for 2 consecutive weeks	[67]
Cerebral ischemia (BCCAO) model mice	Nobiletin prevented associative and short-term memory impairment assessed by passive avoidance and Y-maze. p-CaMKII and MAP2 level, and LTP in the hippocampus were also improved.	50 mg/kg (i.p.) for 7 days before and after carotid artery occlusion	[71]
Cerebral ischemia (MCAO) model rats	nobiletin significantly improved brain edema, neurological deficit score and infarct volume. Nobiletin regulated Akt/CREB/BDNF pathway and Bcl-2 and ameliorated BBB permeability. Nobiletin increased the Nrf2, HO-1, SOD1 and GSH, and decreased the NF-κB, MMP-9 and MDA.	10 and 25 mg/kg (i.p.) pretreatment once daily for 3 days before surgery	[72,73]
Ischemia/reperfusion (t-MCAO) model mice	Nobiletin improved the motor dysfunction, suppressed cerebral edema, infarct volume and apoptosis.	30 mg/kg (i.v.) for before and 1 h after the start of reperfusion	[75]
Ischemia/reperfusion (MCAO) model rats	Nobiletin suppressed the neurological deficits, brain water content and apoptosis via downregulating Bax, caspase3, TNFα, IL6, p-p38 and MAPKAP-2. Nobiletin enhanced the neuroprotective effect of propofol through suppression of Akt/mTOR and NF-kB signaling cascade.	20 mg/kg (i.p.) or 100 mg/kg (p.o.) for 9 days before and an hour prior surgery.	[76,77]
LPS-infused mice	Nobiletin attenuated the LPS-induced microglial activation and memory impairment, and suppressed inflammatory mediators, NO, TNFα, IL-1, and IL-6.	100 mg/kg (p.o.) administration for 6 weeks	[80]
Demyelination model mice	Nobiletin improved the production of oligodendrocyte lineage precursor cells.	50 mg/kg (i.p.) for 3weeks (2 times/week)	[83]
CUMS model mice	Nobiletin improved the immobility time in the tail suspension test and forced swimming test. Nobiletin also suppressed corticosterone level and improved hippocampal BDNF, TrkB, and synapsin I level.	25, 50 and 100 mg/kg (p.o.) for 5 weeks	[86]
BV2 microglia culture system	Nobiletin suppressed TNF-α, IL-1β, p−ERK, p-JNK, and p-p38MAPKs expression.	1-50 μM for 15 min to 24 h	[81]

HuH-7 and 3Y1 cells	Nobiletin suppressed ER stress. Increases *DDIT3*, *TRIB*, and *ASNS* genes and suppressed tunicamycin-induced apoptosis, CCNA2, and TXNIP protein level.	100 μM for 1-4 days	[93,94]
PC12 cells	Nobiletin promoted neurite out-growth, activated PKA/ERK/CREB cascade and CRE-mediated transcription, upregulated NMDA receptor subunits, mAChR, ChAT, CBP and c-Fos proteins, and Inhibited Aβ-induced inflammation.	1-100 μM for 1 min to 24 h	[98,99,100, 107,111,117, 120]
Primary cultured neurons	Nobiletin promoted PKA/ERK/CREB signaling cascade, CRE-dependent transcriptional activity.	1-100 μM for 1 min to 8 h	[39,98,100,1 01,104]
Hippocampal slices	Nobiletin upregulated synaptic transmission by stimulating PKA-mediated phosphorylation of the GluR1.	100 μM for 10-50 min	[104]
SK-N-SH cells	Nobiletin enhanced neprilysin mRNA, protein and its activity.	3-30 μM for 24 h	[118]
iPS cell-derived AD model neurons	Nobiletin reduced intracellular and extracellular Aβ levels, and upregulated neprilysin mRNA levels.	3-30 μM for 24 h	[119]

Aβ; amyloid-β, AchE; acetylcholine esterase, AD; Alzheimer's disease, APP; amyloid-β precursor protein, BBB; blood brain barrier, BCCAO; Bilateral common carotid artery occlusion, BDNF; brain-derived neurotrophic factor, ChAT; choline acetyltransferase, CaMKII; Ca^{2+}/calmodulin-dependent protein kinase II, CREB; cAMP response element binding protein, CBP; CREB-binding protein, CUMS; chronic unpredictable mild stress, DARPP-32; dopamine- and cAMP-regulated neuronal phosphoprotein, ER; endoplasmic reticulum, ERK; extracellular signal-regulated kinase, GDNF; glial cell line-derived neurotrophic factor, IL; interleukin, LPS; lipopolysaccharide, LTP; long-term potentiation, mAChR; muscarinic acetylcholine receptor, MAPKAP-2; MAP kinase-activated protein kinase 2, MCAO; middle cerebral artery occlusion, MPP+; 1-methyl-4-phenylpyridinium, MPTP; 1-Methyl-4-phenyl-1,2,3,6-tetrahydropyridine, mTOR; mammalian target of rapamycin, NF-kB; nuclear factor-kappa B, NMDA; *N*-Methyl-D-aspartate, NO; nitric oxide, OBX; olfactory bulbectomy, PKA; protein kinase A, SAMP8; senescence accelerated mouse-prone 8, t-MCAO; transient middle cerebral artery occlusion, TNFα; tumor necrosis factor-alpha, TXNIP; thioredoxin-interacting protein.

Table S2. Beneficial effect of tangeretin in neurological disease models.

Experimental model	Biological activity	Dosage	Reference
6-OHDA-infused rats	Tangeretin attenuated the 6-OHDA-induced decline in TH-positive cells and the depletion of striatal DA levels.	20 mg/kg (p.o.) for 4 days	[121]
MPTP-infused rats	Tangeretin improved the memory deficits and motor dysfunctions and inhibited dopaminergic degeneration and hippocampal neuronal loss. Tangeretin reduced COX-2, iNOS, IL-1β, IL-6 and IL-2 levels.	50, 100 or 200 mg/kg (p.o.) from 3 days prior to MPTP injection and continued for 20 days	[122]
MPTP/P-injected ATF6α −/− mice	Tangeretin enhanced the expression of UPR-target genes in both dopaminergic neurons and astrocytes, and promotes neuronal survival.	10 mg/kg (p.o.) 24 h and 2 h before MPTP/P injections	[125]
PD model transgenic flies	Tangeretin improved the climbing ability, cognitive deficits and increased in DA content. Tangeretin reduced in various oxidative stress markers, e.g., MAO, TBARS, GST and protein carbonyl content.	5, 10 and 20 μM was added to the diet and the flies were allowed to feed on it for 24 days	[126,127]
Ischemia-reperfusion (MCAO) model rat	Tangeretin decreased brain water content, infarct volume, neurological score, brain edema, and Evans blue leakage. Tangeretin reduced PGE2, iNOS, COX-2, IL-1β, TLR-4 TNF-α, IFN-γ, and IL-6, and oxidative stress markers, GSH, GPx, CAT, GR, MDA, and SOD.	5, 10, 20 mg/kg	[128]
Pilocarpine-infused rat model of epilepsy	Tangeretin reduced the seizure scores and latency to first seizure. Tangeretin improved the pilocarpine-induced suppression of PI3K/Akt signaling and AIF expression. Tangeretin also improved seizure-induced elevations in the activities and expressions of MMP-2 and -9.	50, 100, or 200 mg/kg (p.o.) for 10 days before pilocarpine-injection	[131]
5/6 nephrectomized rats	Tangeretin improved cognitive disturbances and memory impairments via suppress TNF-α, NO, IL-6 and IL-1β, NF-κB/TNF-α/iNOS signaling pathways.	50, 100 or 200 mg/kg (i.g.) for 30 days (starting 5 days after surgery for 35 days)	[135]
Single prolonged stress (SPS)-induced PTSD model rats	Tangeretin improved cognitive impairment. Tangeretin rescued the neurochemical abnormalities and the SPS-induced decreases in DA and 5-HT levels in the hippocampus and amygdala.	100 mg/kg (i.p.) for 14-day after exposure to SPS	[138]

HBMEC cells	Tangeretin improved cell viability in response to OGD-induced injury, and increased the activity of SOD and decrease the levels of ROS and MDA, as well as regulates JNK signaling pathway.	2.5, 5, and 10 μM for 24 h after OGD treatment	[139,140]
Primary cultured rat microglia and BV2 cells	Tangeretin decreased LPS-induced production of NO, PGE_2, TNF-α, IL-1β, IL-6, ROS, iNOS and COX-2. Tangeretin also suppressed LPS-induced NF-kB, HO-1, p-AMPK expression.	30, 50, 100 μM from 1 h prior to LPS for 6-24 h	[142,143]
RASFs	Tangeretin inhibited RASFs proliferation as well as downregulated the expression of MMP-1, MMP-3, COX-2 level and the phosphorylation of ERK, p38 and JNK. Tangeretin also suppressed IL-1-medited NF-κB activation and PGE2 expression.	50, 100 μM for 24-48 h	[144]

5-HT; 5-hydroxytryptamine (serotonin), 6-OHDA; 6-hydroxydopamine, AIF; apoptosis-inducing factor, AMPK; adenosine monophosphate-activated protein kinase, ATF6α; activating transcription factor 6α, CAT; catalase, COX-2; cyclooxygenase-2, DA; dopamine, GPx; glutathione peroxidase, GR; glutathione reductase, GSH; Glutathione, HBMEC; human brain microvascular endothelial cells, HO-1; heme oxygenase-1, IFN-γ; interferon-gamma, IL; interleukin, iNOS; inducible nitric oxide synthase, iPS; induced pluripotent stem, LPS; lipopolysaccharide, MAO; monoamine oxidase, MCAO; middle cerebral artery occlusion, MDA; malondialdehyde, MMP; matrix metalloproteinase, MPTP; 1-Methyl-4-phenyl-1,2,3,6-tetrahydropyridine, MPTP/P; MPTP/probenecid, NF-kB; nuclear factor-kappa B, NO; nitric oxide, OGD; oxygen-glucose deprivation, PI3K; Phosphoinositide 3-kinase, PGE_2; prostaglandin E_2, PTSD; post-traumatic stress disorder, TLR-4; Toll-like receptor 4, TNFα; tumor necrosis factor α, RASFs; rheumatoid synovial fibroblasts, SOD; superoxide dismutase, TBARS; 2-thiobarbituric acid reactive substances, TH; tyrosine hydroxylase, UPR; unfolded protein response.

Table S3. Multiple effects of 3,3',4',5,6,7,8-heptamethoxyflavone (HMF) in neurological disease models.

Experimental model	Biological activity	Dosage	Reference
Cerebral ischemia (CCAO) model mouse	HMF improved memory impairment and neuronal death via increases in p-ERK1/2, p-CAMKII and p-CREB level, and suppresses microglial activation in the hippocampus after ischemia. HMF enhanced BDNF and Dcx expression in the hippocampus.	25 or 50 mg/kg (s.c.) for 7days	[147,148]
MK-801-infused mice	HMF improved MK-801-induced spatial memory impairment and locomotive hyperactivity by activating ERK signaling in the hippocampus and cortex.	50 mg/kg (s.c.) for 7 days	[149,150]
LPS-injected mice	HMF suppressed LPS-induced losses in body weight, microglial activation, IL-1β, TNFα, COX-2 expression in the hippocampus.	100 mg/kg (s.c.) for 1-10 days	[151]
Corticosterone-induced depression model mice	HMF prevented corticosterone-induced reductions in BDNF level in the hippocampus. HMF ameliorates corticosterone-induced reductions in neurogenesis, as well as p-CaMKII and p-ERK level in the hippocampus.	50 mg/kg (s.c.) 9, 16, or 25 days	[153]
CUMS-model mice	HMF ameliorated CUMS-induced depressive-like behavior through rescues reduction in BDNF, p-CaMKII, p-ERK levels and neurogenesis in the hippocampus.	50 or 100 mg/kg (p.o.) for 15 days	[154]
Primary cultured cortical neurons	HMF activated ERK/CREB cascade in cultured neurons.	1-100 μM for 10-90 min	[149]
Primary cultured astrocytes	HMF decreased LPS-induced iNOS, p-p38 levels and NF-kB activation.	25-100 μg/ml for 30 min-24 h	[152]
C6 cells	HMF inhibited PDE4B and PDE4D activity, and enhanced cAMP, p-ERK and p-CREB level, and BDNF expression.	10 μM for 48 h	[155]

BDNF; brain-derived neurotrophic factor, CaMKII; Ca2+/calmodulin-dependent protein kinase II, CCAO; bilateral common carotid artery occlusion, COX-2; cyclooxygenase-2, CREB; cAMP response element binding protein, CUMS; chronic unpredictable mild stress, Dcx; doublecortin, ERK;

extracellular signal-regulated kinase, IL; interleukin, iNOS; inducible nitric oxide synthase, LPS; lipopolysaccharide, NF-kB; nuclear factor-kappa B, PDE; phosphodiesterase, TNFα; tumor necrosis factor-alpha,
